# Large-scale manufacturing of precisely patterned flexible soft tissue implants with high porosity

**DOI:** 10.1088/1758-5090/ae7697

**Published:** 2026-06-15

**Authors:** Amal Shabazz, Alexandra P Christensen, David Garvey, Mike Pallotta, Sahar Vakili, Stephen Farias, John P Fisher

**Affiliations:** 1 Fischell Department of Bioengineering, University of Maryland, College Park, MD, United States of America; 2 Center for Engineering Complex Tissues, University of Maryland, College Park, MD, United States of America; 3 The LaunchPort, Baltimore, MD, United States of America; 4 Materic, Baltimore, MD, United States of America

**Keywords:** 3D printing, Formlabs, stereolithography, tissue engineering, good manufacturing practice

## Abstract

As improvements in material compatibility are achieved, stereolithography (SLA) 3D printing allows for the prototyping of soft tissue constructs with high reproducibility and precision. To streamline the Food and Drug Administration approval process, clinical-grade manufacturing must be validated. Here, we present a manufacturing process and post-manufacturing analysis for soft-tissue implants used to reconstruct the nipple-areolar complex (NAC). The NAC is a specialized soft tissue structure that influences aesthetic outcomes in breast reconstruction. Physiologically-sized NAC implants were designed and printed in a good manufacturing practice facility using Formlabs Biomed Elastic 50 A (E 50 A) and Biomed Flexible 80 A (F 80 A) resins. Documentation and procedures were established to standardize and control macroporous soft-tissue implant manufacturing. Post-manufacturing classification based on architectural and mechanical features defined the minimum acceptable criteria for future production. E 50 A macroporous implants resembled the modulus of soft tissue, while F 80 A geometries showed higher reproducibility and preservation of fine architectural features. SLA printing enabled the patterning of implants with porosities of 74.65% and 72.74% for 0.4- and 0.5- mm geometries, respectively. 3D printed implants supported the infusion of gelatin methacryloyl for the delivery of biological cues, with a filling efficiency greater than 97%. This manufacturing platform achieves reproducible fabrication of soft-tissue implants that meet both structural and mechanical requirements. Further, the presented methodology can be adapted for a wide range of complex soft tissues to advance regenerative medicine solutions toward regulatory approval and eventual clinical integration.

## 1. Introduction

The ability to fine-tune architectural features holds great promise for tissue engineering and regenerative medicine, enabling the reproducible construction of identical or patient-specific scaffolds. In soft tissue engineering, the challenge lies in balancing the polymeric properties needed for photopolymerization with the biological cues and mechanical strength inherent to elastic tissues [1]. Stereolithography (SLA) can be used for 3D printing of structures with elastic-like properties. SLA offers several advantages over traditional filament-based fabrication methods, including the construction of repeatable, complex geometric features and high-resolution down to 25 *μ*m [2]. Additionally, SLA technology can be used at a mass production scale, thereby printing batches of soft tissue constructs rather than printing them individually. While there are currently no Food and Drug Administration (FDA) regulations for the approval of 3D printed implants, the development of advanced biomaterial-based 3D printed products intended for implantation requires clinical-grade manufacturing validation [3–5].

Acellular 3D printed soft tissue scaffolds intended for implantation fall under the classification of Class II or Class III medical devices [6]. They are thus regulated through pre-market approval or 510(k) pre-market notification pathways, depending on the existence of a similar product. Detailed documentation of technical, preclinical, and clinical data must be submitted to the FDA for approval. Section 820 of the FDA’s CFR 21 details the good manufacturing practice (GMP) requirements to manufacture, package, and store medical products under a quality management system. Furthermore, the international standard ISO 13845 encompasses and builds upon GMP principles, providing guidance surrounding risk stratification and product lifecycle support for medical device manufacturing. To better analyze SLA’s viability for GMP manufacturing, our team partnered with an FDA-registered, ISO 13485-certified medical device manufacturing facility. We developed preliminary process guidelines and quality mechanisms for soft tissue manufacturing and executed them in trial builds that emulated a qualified GMP production line.

The large-scale fabrication of soft tissue implants that balance high porosity and high-resolution features is unprecedented in this field. Uniform porosity is an essential factor for cell attachment, proliferation, and function [7]. Adequate nutrient exchange is necessary for migrating cells to support vascularization and healthy tissue ingrowth. Tissue engineering solutions in this area are either focused on the use of rigid, bioresorbable implants with fine features for printing hard tissues such as bone, or on the use of deformable, gel-like biomaterials with low resolution for printing soft tissues such as skin. Scaffolds with large, interconnected pores significantly enhance cellular migration, tissue formation, and vascularization, thereby improving long-term healing outcomes [8].

Despite these promising advances in scaffold design and manufacturing, applying these technologies to clinically relevant soft tissue engineering solutions remains a significant challenge. The nipple-areolar complex (NAC) is a key example of delicate, specialized soft tissue that plays an important aesthetic role in breast reconstruction. Our previous work pioneered the development of a porous extrusion-printed NAC using a two-material solution that maintains long-term projection while aiding dermal regrowth into the implant [9]. While this work shows our ability to restore nipple shape while integrating with surrounding tissue, improvements in reproducibility are needed to facilitate clinical translation. SLA can be used to manufacture the structural, macroporous component of a NAC implant that can be filled with a hydrogel to encourage tissue integration or impart bioactivity.

The goal of this study is to engineer a large batch of 3D-printed soft tissue NAC implants and pilot their manufacturing processes within an ISO 13485-certified facility. Our first objective was to create a manufacturing process plan (MPP) comprising work instructions, inline inspection documents, and product inspection procedures to standardize and control macroporous NAC manufacturing. The second objective was to assess the architectural and mechanical characteristics of the 3D printed implants to establish initial acceptance criteria for repeatable manufacturing. As initial validation of the materials and quality process, our final objective was to verify the feasibility of the implant design in providing structural and biological support. Using a combination of additive manufacturing design software and prototyping methods, we designed and prototyped SLA printed flexible NACs with interconnected and structurally stable large pores. The GMP process was outlined and documented to optimize future runs. This technology can be applied broadly to soft tissue engineering for the batch manufacturing of macroporous, implantable tissue constructs.

## 2. Materials and methods

### 2.1. Implant design

Macroporous NAC implants were designed in a CAD software (SolidWorks) to match human physiological dimensions of 10 mm nipple height, 10 mm nipple diameter, 24 mm areolar diameter, and 2 mm areolar height. Implants were embedded with internal porosity using an additive manufacturing software (Netfabb, AutoDesk) to pattern internal geometries. A variety of infill patterns were tested and analyzed based on printability, reproducibility, and mechanical strength before narrowing down to the grid pattern. Parts were designed with a 0.4- or 0.5- mm strut diameter at 15% density with a 0.5 mm outer shell. Pores remained consistent throughout the height of the structure. An engineering drawing was created in SolidWorks for each geometry. Once optimized, designs were sliced in the commercially available slicing software (Preform, Formlabs) and sent to the GMP facility for manufacturing. Implants were printed using the SLA printer (Form 3BL, Formlabs) to prototype 40 samples of each geometry with Formlabs BioMed Elastic 50 A (E 50 A) or BioMed Flexible 80 A (F 80 A) resin in the GMP facility.

### 2.2. Quality management for GMP

Prior to printing, a detailed procedure was drafted that outlined instructions for printing and post-processing based on the specified implant design. A Line Preparation and Clearance Inspection Form was prepared and completed to indicate print readiness. Detailed work instructions were outlined to identify the equipment, materials, fabrication, post-processing, and packaging guidelines for manufacturing the implants. The outer packaging of all materials was thoroughly examined for defects, and a materials receipt and specification record was created. A comprehensive traveler document was also developed to guide inline inspections and ensure material traceability. Technical documents were prepared detailing acceptance criteria to guide outgoing inspections. A total of 20 samples were oriented on the build plate for each run, with two runs per part design. Samples were post-processed according to the manufacturer’s recommendations. Briefly, parts were washed in 99% isopropyl alcohol for 20 min and then air-dried for at least 30 min. Implants were then UV-crosslinked for 30 min at 70 °C in a transparent container filled with deionized water. Supports were carefully removed with a sterile razor utility knife.

### 2.3. Pilot run management

A Production Report was completed to document the processing specifications for each run, including the lot and manufacturing order number, resin volume and cartridge number, resin tank number and print duration, and the process start and end dates. For each step, the operator initialed critical processes before continuation. An Inspection Report was completed to assess the quality of three randomly selected parts after production. Accepted parts were placed into a sterile bag and labeled with the part number, production lot number, and sample number. Implants that failed inline and/or outgoing inspections (e.g. with splits, bubbles, severely broken struts, warping, or major deformities) were quarantined as non-conforming materials (NCM) with associated NCM reports. These parts underwent further assessment to quantify acceptance vs rejection features. Throughout the process, the operator completed a manufacturing report at each step and had it verified by the site’s quality manager. First article out-of-specification criteria were outlined based on part dimensions, visual appearance, and architectural characteristics to improve future runs.

### 2.4. Post-handling physical characterization

Upon receiving the samples, the mass was recorded for 10 samples of each geometry on a mass balance. The physical dimensions of 40 samples were measured using digital calipers. Acceptance criteria were defined as the mean ± 2x the standard deviation. The integrity of the base layer of pores was assessed with brightfield microscopy (Eclipse Ti2, Nikon). A large image was taken of the inferior layer of the NAC for each print with an exposure time of 1 s. Strut diameter was measured in ImageJ by averaging the length of ten individual struts within a given print layer for 5 samples of each geometry.

### 2.5. Mechanical testing

An Instron 5942 was used to perform compression testing of the 3D printed NACs. A total of 10 samples of each of the four geometries were tested, along with solid NACs with no infill patterning for both materials as controls. Samples were compressed at a rate of 1.3 mm min^−1^, and the construct effective modulus was calculated using the initial linear portion (0.1–0.5 mm displacement).

A 4-0 monofilament nylon suture (Ethilon) with an FS-2 19 mm 3/8 c reverse cutting needle was used to create a suture loop in 3 NACs for each of the four different print groups. The suture was inserted 2 mm from the edge of the areola and tied in a loop around a 3D printed tensile grip attachment. Tensile testing was performed using an Instron 5942. The opposite side of the sutured NAC was clamped in the bottom tensile grip. The testing displacement rate was set to 1 mm min^−1^, and the specimen was pulled until the suture broke through the printed NAC. The force at break, or suture retention strength, was recorded.

### 2.6. MicroCT imaging

Samples were scanned with a Bruker Skyscan 1276 (Bruker, Germany) at 100 kV, 200 *µ*A, and 40.7 *µ*m pixel size. Rotational images were reconstructed into 2D slices with NRecon (Bruker, Germany). Cross-sectional slices were extracted in Data Viewer software (Bruker, Germany). The area of individual coronal slices was measured in ImageJ. Porosity was calculated as the ratio of void area to total area (void area + solid area) for each cross-section of struts.

### 2.7. Hydrogel filling

Gelatin methacryloyl (GelMA) was synthesized according to a modified version of a previously established protocol [10]. Briefly, Type A porcine skin gelatin (300 bloom; Sigma-Aldrich) was dissolved at 10% (w/v) into phosphate-buffered saline (PBS; Thermo Fisher Scientific) at 50 °C for 20 min. Methacrylic anhydride (Sigma-Aldrich) was added dropwise to the gelatin solution under vigorous stirring for 3 h (0.6 g MA per gram of gelatin). The mixture was diluted 1:1 with PBS to stop the reaction and centrifuged at 1000 g for 2 min. The supernatant was collected and dialyzed in deionized water (10 kDa molecular weight cutoff; Thermo Fisher Scientific) for 7 d to remove excess salts and acids. The dialyzed GelMA was then adjusted to a pH of 7.3–7.5 and frozen at −80 °C before lyophilization for 7 d. This procedure yielded a porous foam that was stored at room temperature until further use. The lyophilized product was reconstituted in PBS at 5% w/v% and pipetted into the NAC from the areolar opening. Lithium phenyl-2,4,6-trimethylbenzoylphosphinate (LAP, Tocris) was added as a photoinitiator at a concentration of 0.1% w/v. Constructs were UV-crosslinked for 2 min before MicroCT imaging. The filling percentage was determined as the ratio of solid area to total area for each cross-section of struts.

### 2.8. Statistical analysis

Statistical analyses were conducted in GraphPad Prism. Data is presented as mean ± standard deviation. A Shapiro–Wilk test was used to test for normal distribution. Normal datasets were evaluated with Student’s *t*-tests and one-way ANOVA tests. Groups that were found to be significantly different were then further analyzed through post hoc comparisons using Tukey’s test. Datasets with non-normal distribution were tested using Kruskal–Wallis test with Dunn’s multiple comparisons test. Statistical significance was indicated by *p < 0.05, **p < 0.01, ***p < 0.001, and ****p < 0.0001.

## 3. Results

### 3.1. Implant design and GMP development

**Figure 1.**
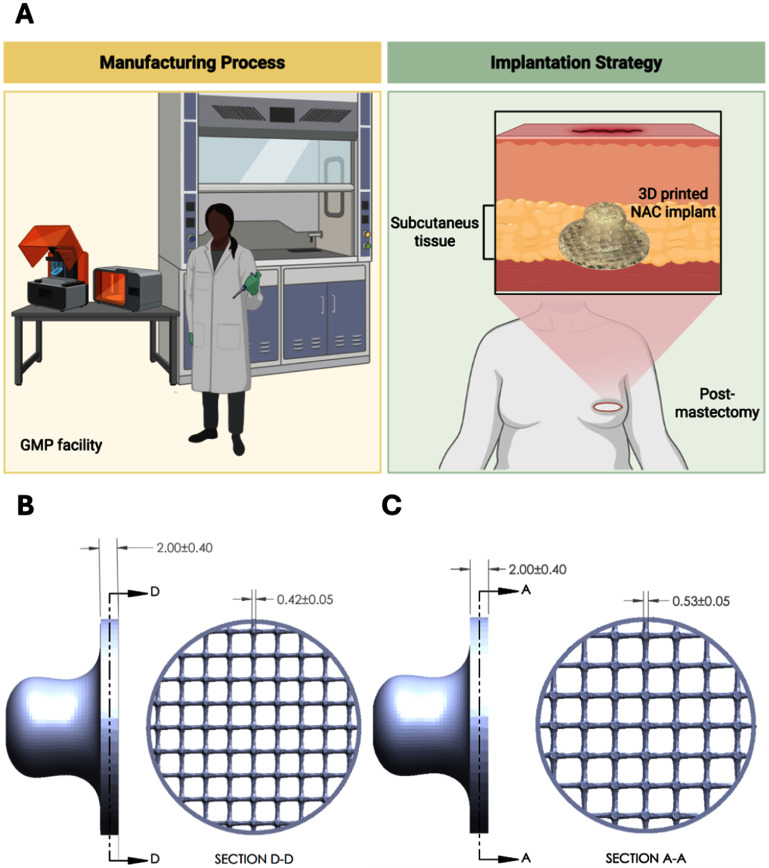
Physiologically-sized 3D printed NAC implants were designed with macroporous internal structure. (A) Prototyping and implantation strategy for 3D printed NAC implants. Implants are manufactured following GMP protocols to ensure the safety and efficacy of the product. NACs are envisioned to be integrated with current clinical breast reconstruction procedures post-mastectomy. Created in BioRender. Shabazz, (A). (2026) https://BioRender.com/ermpf5y. Engineering drawing detailing design parameters and tolerances for (B) 0.4 mm and (C) 0.5 mm geometries. A large batch (*n* = 160) of macroporous implants was printed using Formlabs Biomed E 50 A or F 80 A in a GMP facility before architectural and mechanical assessment.

Implants were designed to resemble the human anatomical nipple with a nipple height of 12 mm, an areola diameter of 24 mm, and a nipple diameter of 10 mm. CAD-generated designs of implants measured a nipple height of 12.56 ± 0.35 mm, an areola diameter of 23.98 ± 0.3 mm, and a nipple diameter of 10.96 ± 0.75 mm (figure 1). Implants were patterned with a grid internal structure consisting of 0.42 ± 0.05 or 0.53 ± 0.05 mm diameter struts. Implants were designed with a 2.0 ± 0.40 mm areola height to facilitate suturing of the implant onto the patient in the long term.

**Figure 2.**
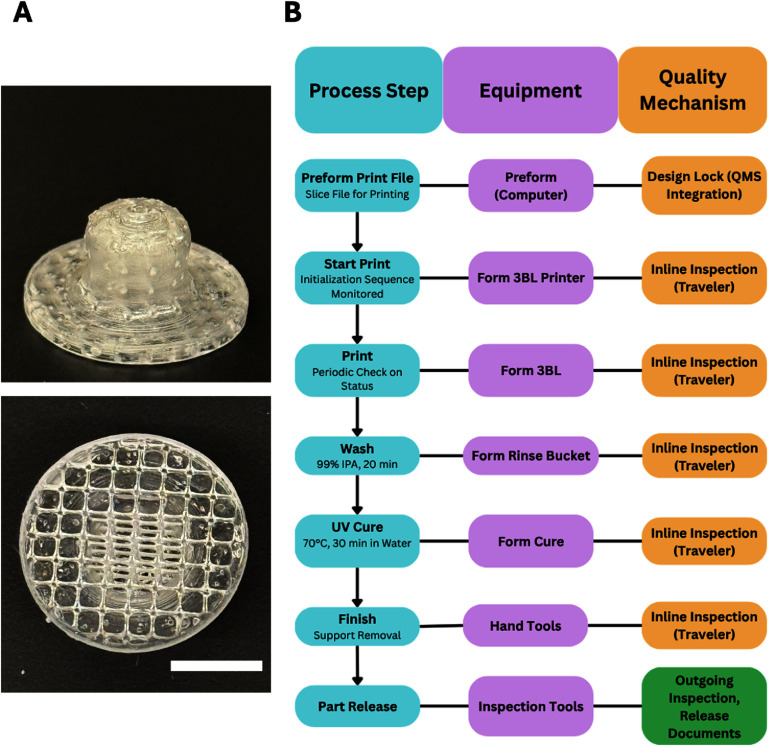
Grafts are manufactured under GMP specifications. (A) Representative images of physiologically sized 3D printed NACs with macroporous architecture. Implants were printed with E 50 A or F 80 A material with hollow internal porosity in a grid pattern. Scale bar represents 1 cm. (B) Flow chart of GMP process development outlining processing, equipment, and quality control parameters performed for the prototyping, inspection, and release of implants.

Implants were fabricated in a GMP facility, producing 40 BioMed Elastic 50 A 0.4 mm (E 0.4), 40 BioMed Elastic 50 A 0.5 mm (E 0.5), 40 BioMed Flexible 80 A 0.4 mm (F 0.4), and 40 BioMed Flexible 80 A 0.5 mm (F 0.5) implants with an internal grid structure. Of those produced, 11 implants failed QC inspection and were quarantined as NCM. A total of 149 constructs were processed for subsequent architectural and mechanical analysis (figure 2).

Manual measurements of the physical dimensions of printed implants in table 1 showed a construct height of 12.09 ± 0.25 mm, nipple diameter of 10.85 ± 0.26 mm, areola diameter of 24.26 ± 0.18 mm, and areola height of 2.26 ± 0.10 mm. These results indicate that the printed implants were within the tolerance ranges of the designed structures and that the robust precision of SLA printing preserves print fidelity and quality, making it optimal for future GMP production. NAC architectural features were accurately and reproducibly printed. Criteria for architectural, mechanical, and visual inspection characteristics were defined as a baseline for future runs.

**Table 1.** Process flow criteria with acceptable process capabilities for future run optimization. Data are reported as mean ± 2x the standard deviation, where *n* = 40.

Test	Specification
Physical characteristics	
Total height (mm)	12.09 ± 0.489
Nipple diameter (mm)	10.85 ± 0.528
Areola diameter (mm)	24.26 ± 0.361
Areola height (mm)	2.26 ± 0.203

Architectural characteristics	
Broken struts (n)	9.8 ± 6.32
Infill density (%)	24.07 ± 9.58
Compressive modulus (MPa)	E 50 A: 0.505 ± 0.272 F 80 A: 3.774 ± 0.968

Visual Inspection characteristics	
Discoloration	Not detected
Dust	Not detected

In addition to establishing baseline implant production criteria, an MPP was established. Work instructions, inline inspection documents, and product inspection procedures were outlined to monitor implant geometry and material requirements (**supplemental 1**).

### 3.2. Mechanical analysis demonstrates resemblance to soft tissue

**Figure 3.**
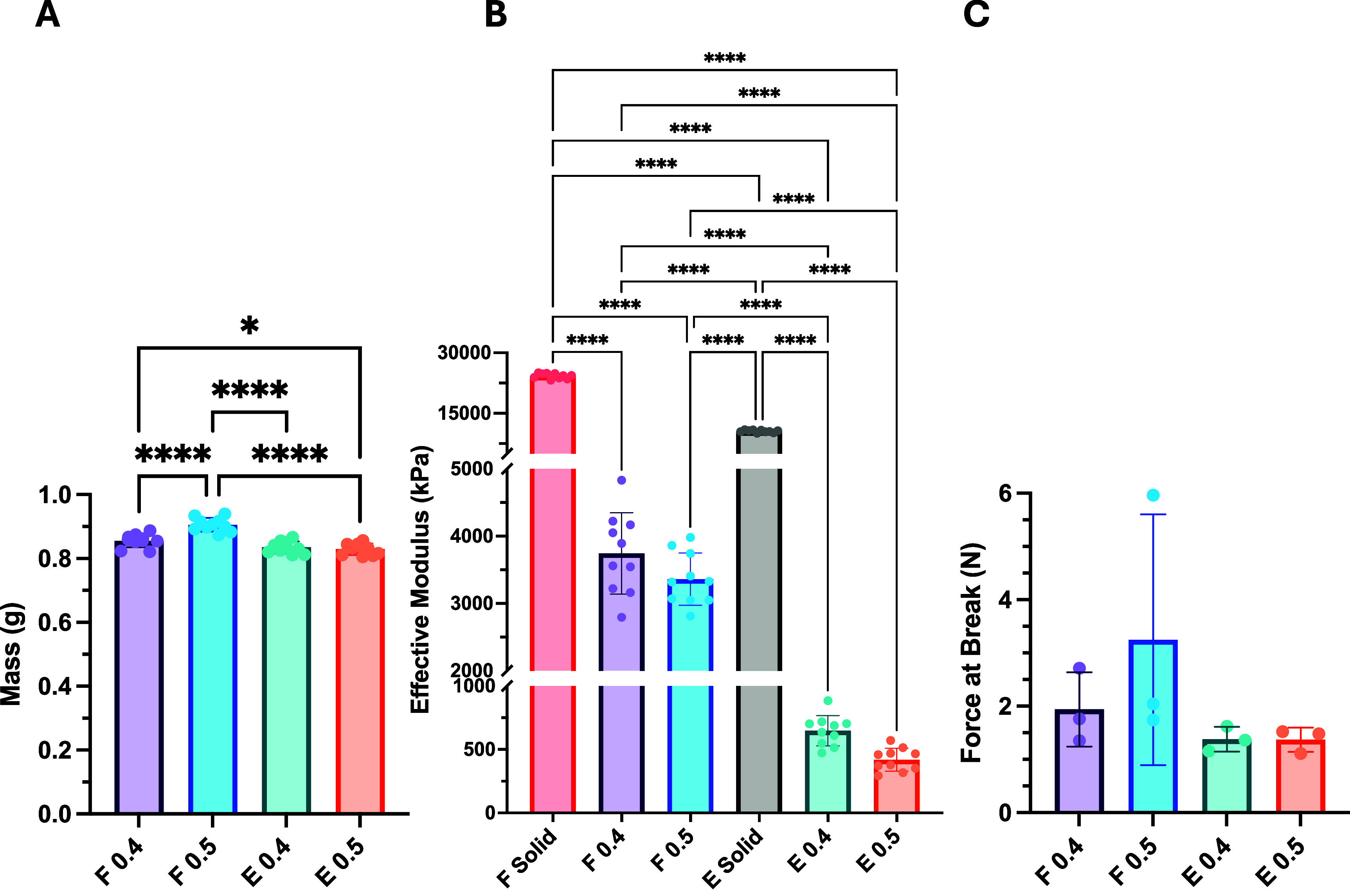
Mechanical properties of 3D printed NAC implants resemble that of soft tissue. (A) Recorded mass of samples of each design. Physiologically sized implants weighed under 1 g. More variation was observed in F 80 A designs. Little variation was observed between samples, demonstrating the reproducibility achieved through SLA printing. *n* = 10 for each group. *p < 0.05, ****p < 0.0001. (B) Effective modulus was measured through uniaxial compression testing, *n* = 10 for each group. ****p < 0.0001. (C) Suture retention strength for 3D printed NAC implants. *n* = 3 for each group. Data was analyzed using single-factor analysis of variance (ANOVA) followed by Tukey’s multiple comparison test assuming normal data distribution with a confidence of 95% (*p* < 0.05).

NAC design and post-processing technique, specifically print support removal, impact the final mass of the individual implants. While all measured implants were between 0.81 g and 0.94 g (figure 3(A)), F 0.5 NACs had the highest mass (****p < 0.0001). No statistically significant differences were seen between E 0.4 and E 0.5 mm designs. F 0.4 and E 0.4 mm geometries also showed no statistically significant differences.

One desired outcome of 3D printing NACs is to replicate the elasticity of natural tissue. Compression testing was used to evaluate the effective modulus of F 80 A and E 50 A NACs (figure 3(B)). As expected, decreasing the infill density to 25% from a solid structure resulted in a significant decrease in modulus (****p < 0.0001). No statistical significance was shown between NAC moduli when varying the strut diameter. Modulus varied between materials, as F 80 A designs had significantly higher modulus than their E 50 A counterparts (****p < 0.0001).

As these implants are intended for surgical implantation, sutures may be used to secure the NAC in place. Using a suture retention test, or suture pulls, verifies that the material’s properties withstand the suturing process during implantation. No statistically significant differences were seen between materials or between any designs (figure 3(C)).

### 3.3. Architecture is preserved throughout the construct

**Figure 4.**
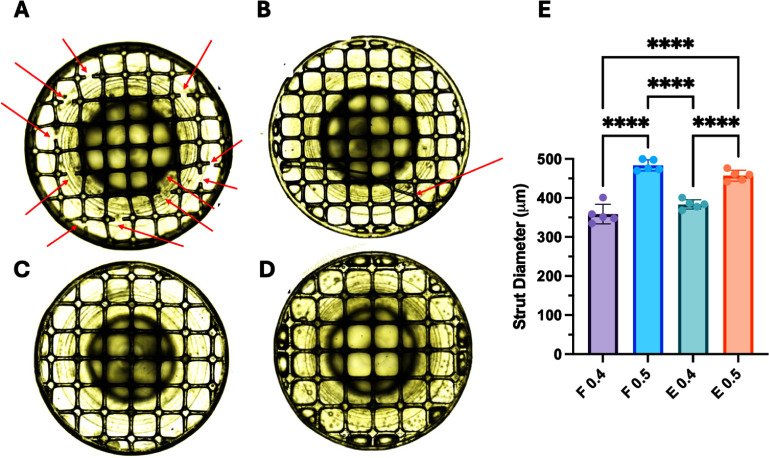
Architectural integrity varies by design. Brightfield images of the inferior layer of 3D printed NAC with (A) E 50 A 0.4 mm, (B) F 80 A 0.4 mm, (C) E 50 A 0.5 mm, and (D) F 80 A 0.5 mm geometries. Arrows indicate cosmetic defects from broken struts or printed artifact. (E) Strut diameter measurements for each geometry. *n* = 5 for each group, *n* = 10 technical replicates. ****p < 0.0001. Data was analyzed using single-factor analysis of variance (ANOVA) followed by Tukey’s multiple comparison test assuming normal data distribution with a confidence of 95% (*p* < 0.05).

To explore the impact of the printing and post-processing workflow on NAC integrity, we sought to visually quantify defects on the implant’s inferior surface using brightfield microscopy. Strut diameter was measured across the bottom layer of the infilled pattern and quantified (figure 4). The printed geometries were within tolerable limits of the engineering design, with the 0.4 mm geometries reporting an average strut diameter of 383.3 ± 11.85 *μ*m and 358.7 ± 25.12 *μ*m for E 50 A and F 80 A, respectively. Similarly, the 0.5 mm geometries reported strut diameters of 457.1 ± 13.78 *μ*m and 483.4 ± 14.16 *μ*m for E 50 A and F 80 A. The low variability in these measurements demonstrates the robust manufacturability of these implants for large-scale production.

**Figure 5.**
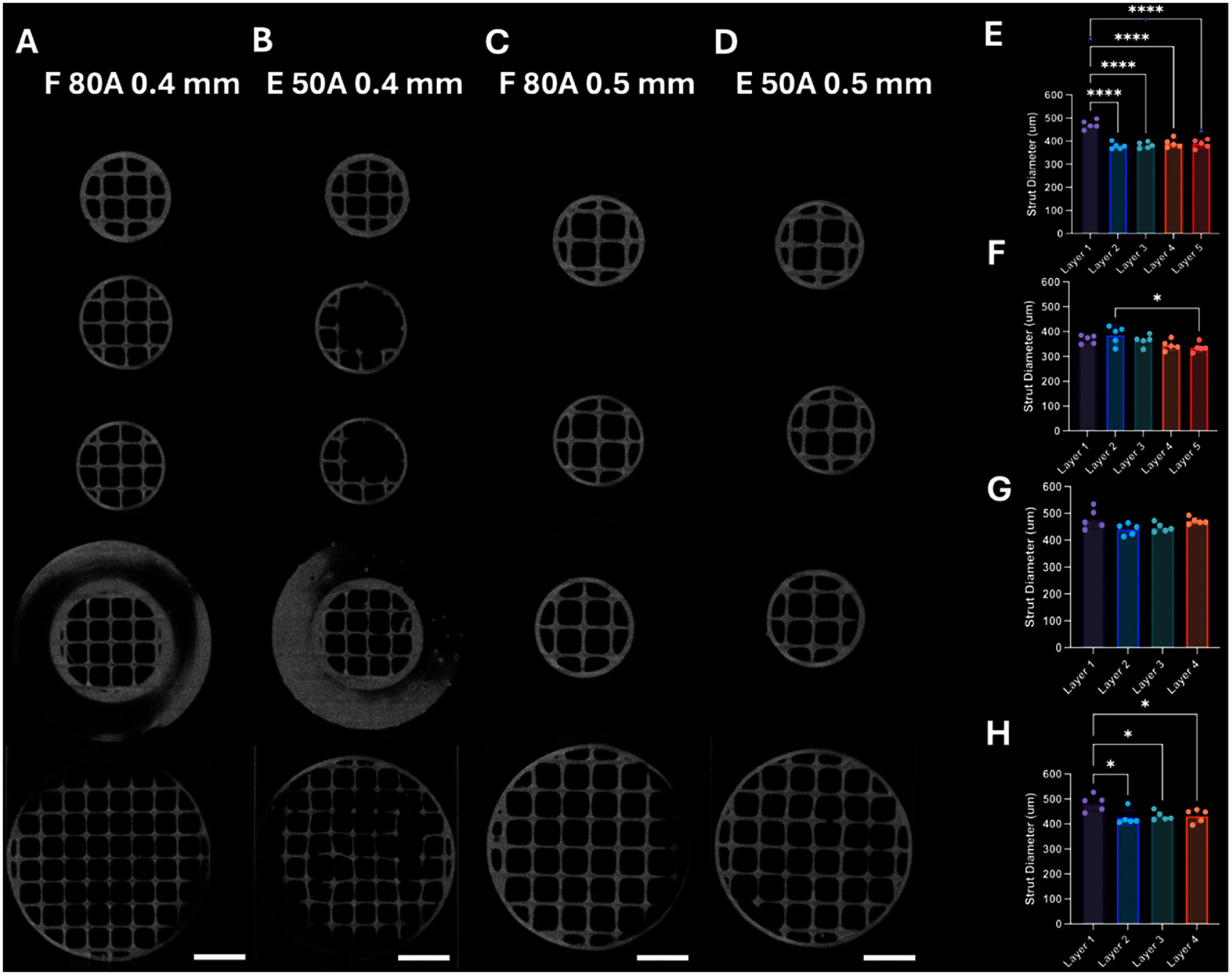
Print integrity is maintained throughout the print height. (A) Cross-sectional Micro-CT images of each layer in F 80 A 0.4 mm, (B) E 50 A 0.4 mm, (C) F 80 A 0.5 mm, and (D) E 50 A 0.4 mm geometries. Strut diameter measurements from Layer 1 (top) to Layer 5 (bottom) for (E) F 80 A 0.4 mm, (F) E 50 A 0.4 mm, (G) F 80 A 0.5 mm, and (H) E 50 A 0.4 mm geometries. Scale bar represents 7 mm. *p < 0.05, ****p < 0.0001. *n* = 5 for each group, *n* = 10 technical replicates. Data was analyzed using single-factor analysis of variance (ANOVA) followed by Tukey’s multiple comparison test assuming normal data distribution with a confidence of 95% (*p* < 0.05).

We next sought to assess the consistency in the designed pattern throughout the height of the structure. Implant stability and structural integrity were further measured through MicroCT imaging to visualize the internal porosity throughout the print (figure 5). The F 80 A 0.5 mm geometry showed the least variation in architectural features between each layer. For each other geometry, the top layers had a higher strut diameter than the subsequent layers.

**Figure 6.**
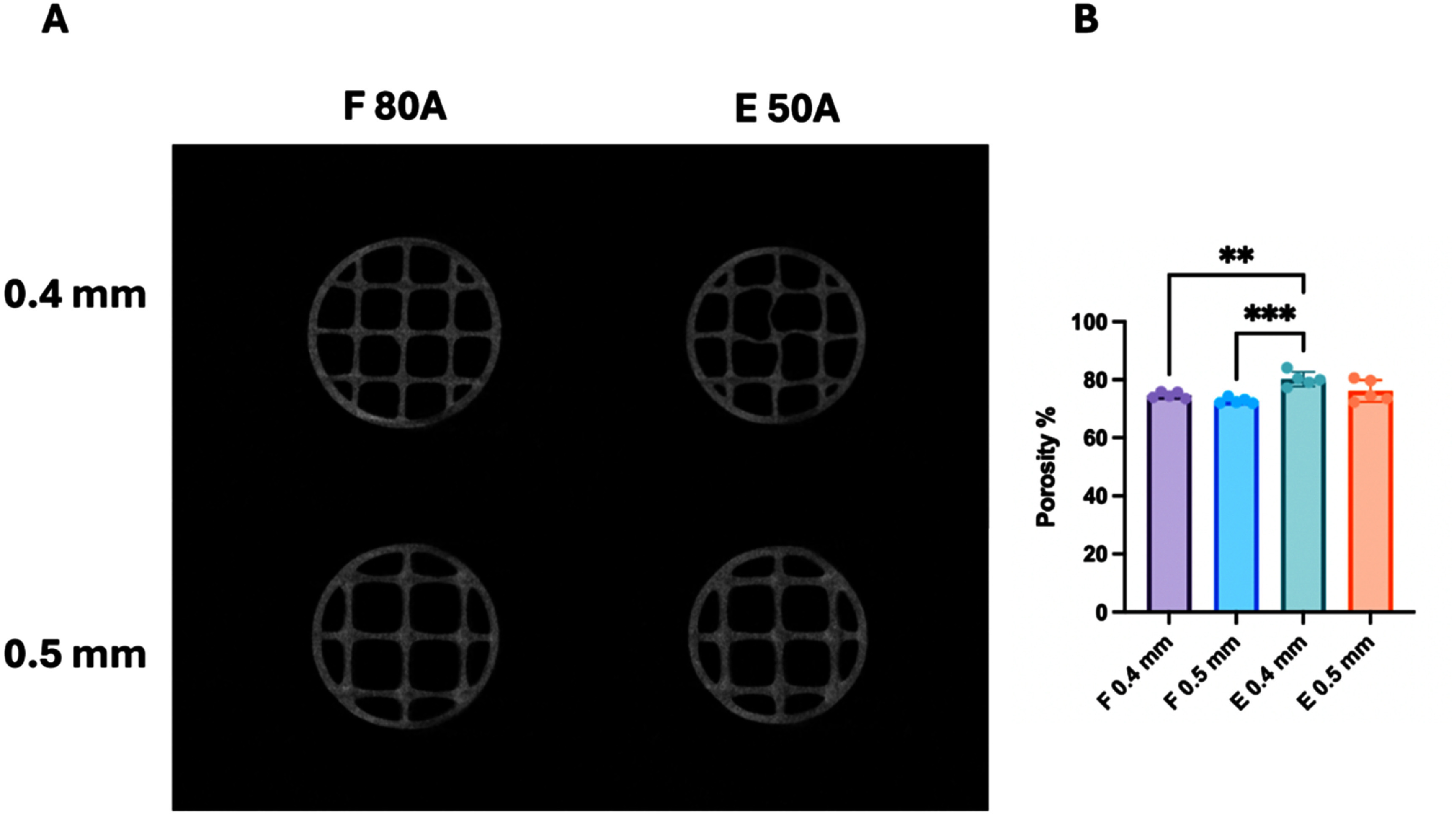
Macroporous architecture can accommodate tissue invasion. (A) Cross-sectional Micro-CT images of empty NAC implants visualizing macroporous architecture. (B) Quantification of void pore area for each geometry. **p < 0.01, ***p < 0.001. *n* = 5 for each group. Data was analyzed using single-factor analysis of variance (ANOVA) followed by Tukey’s multiple comparison test assuming normal data distribution with a confidence of 95% (*p* < 0.05).

To prevent nipple flattening and ensure reproducibility, the infilled pattern must be consistent across each layer and withstand remodeling or deformation during long-term implantation. To test the consistency of architectural features across the height of the print, we quantified the porosity in each layer of the implant (figure 6). The consistency in total porosity calculated from MicroCT images was higher for F 80 A prints, measuring 74.65% and 72.74% for 0.4 and 0.5 mm geometries, respectively. While the prints were designed with a 15% infill density, a higher solid area percentage was observed, likely due to the large node size at strut junctions. Higher variation was observed in E 50 A prints, where porosity was 80.20% and 76.13% for 0.4 and 0.5 mm geometries, respectively.

### 3.4. Implants facilitate efficient hydrogel delivery

**Figure 7.**
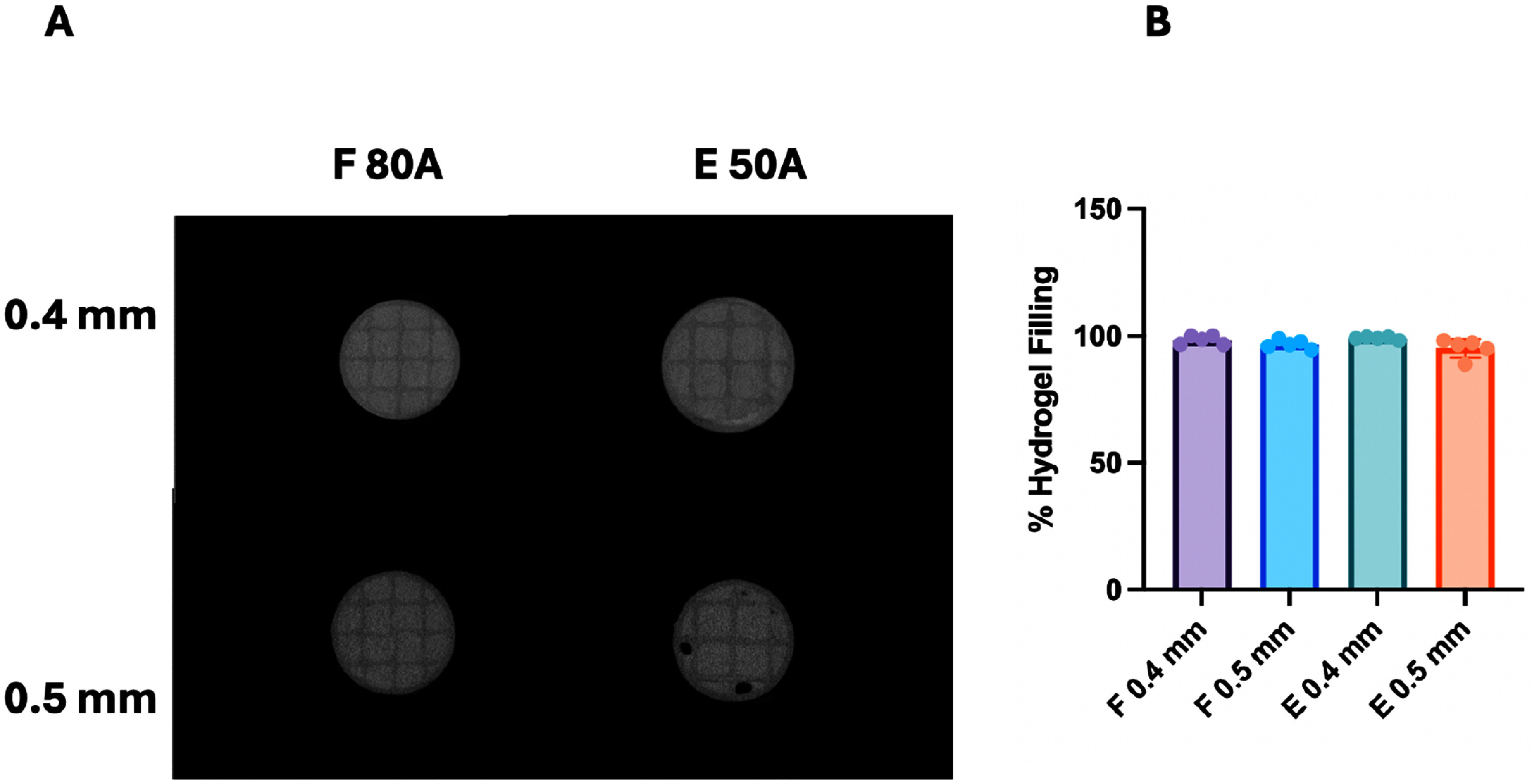
Grafts are efficiently filled with GelMA hydrogel. (A) Micro-CT images of GelMA-filled NAC implants. Hydrogel infusion signifies the ability to deliver biochemical cues to infiltrating cells upon implantation. (B) Quantification of hydrogel filling efficiency for each geometry. *n* = 5 for each group. Data was analyzed using single-factor analysis of variance (ANOVA) followed by Tukey’s multiple comparison test assuming normal data distribution with a confidence of 95% (*p* < 0.05).

The filling efficiency of NAC implants was assessed by injecting a photocurable hydrogel solution into the macropores. GelMA was selected due to its favorable biocompatibility, tunability, and manufacturability. MicroCT images show an even distribution of GelMA solution throughout the scaffold (figure 7(A)), with the hydrogel occupying 98.33%, 99.12%, 95.15%, and 96.71% of the implant area for F 0.4, E 0.4, E 0.5, and F 0.5 mm geometries, respectively (figure 7(B)). These results reveal that GelMA was well dispersed throughout the implant, highlighting the potential to incorporate biological cues embedded within the implanted NAC.

## 4. Discussion

In this study, we established the production of physiologically-sized, GMP-grade 3D printed flexible soft tissue implants with large, interconnected pores. Soft tissue regeneration remains a clinical reconstructive challenge due to the intricate hierarchical organization and complex mechanics of native tissue [11, 12]. Vat photopolymerization technologies are increasingly used in tissue engineering applications for precise geometric control and to impart porosity in scaffolds. The combination of an elastic synthetic polymer for structural support and a degradable hydrogel for delivery of bioactive cues is a promising approach to engineer soft tissues. While there are no current FDA guidelines on implants produced with additive manufacturing, a thorough analysis of design quality is necessary to initiate such approval. This analysis was conducted with three objectives: first, to classify documentation and procedures to standardize and control macroporous NAC manufacturing; second, to assess the architectural and mechanical characteristics of the 3D printed implants; and third, to verify the feasibility of the implant design in providing structural and biological support.

Macroporous implants have been shown to encourage new tissue growth upon implantation in hard tissues [13–15]. However, studies of the influence of macroporous architecture in 3D printed soft tissue implants intended for implantation are limited. Because product quality varies across manufacturing technologies, parameters, and process steps, assessing the variability of each input parameter is critical for building reproducible parts. Feature geometry, overall dimensions, material characteristics, and mechanical properties must be outlined, with design validation occurring before mass production [16]. Our findings demonstrate that material properties and geometric design play a large role in determining the mechanical and architectural integrity of the implants. A large batch of 160 implants was printed using Formlabs technology, and the GMP manufacturing process was documented to outline manufacturing capabilities (figure 2(B)). Process development criteria were outlined to determine process limitations for baseline manufacturing capabilities.

Acellular implants were chosen for this analysis to 1) streamline the FDA approval process, 2) expedite design and *in vitro* testing, and 3) reduce experimental cost. We focused on improving the reproducibility, manufacturability, and feasibility of 3D printed NACs. SLA printed soft elastomeric implants were infused with a hydrogel to deliver biological cues and encourage tissue infiltration upon implantation.

Formlabs BioMed materials are manufactured in an FDA-registered, ISO 13485-certified facility, following stringent protocols to ensure consistency and manufacturability. Both E 50 A and F 80 A are verified as biocompatible for processes requiring long-term skin contact (>30 d) and short-term mucosal membrane contact (<24 h), in accordance with ISO 10 993 and USP Class VI standards. These materials are sterilizable through gamma/electron beam irradiation or ethylene oxide but are not suitable for autoclave treatment. E 50 A is a soft elastomeric resin with a reported ultimate tensile strength of 2.3 MPa and a 150% elongation at break. F 80 A is a firm, but flexible material with a reported ultimate tensile strength of 7.2 MPa and 135% elongation at break. While the majority of consumers use these products for tissue models or flexible medical devices [17], we see potential for the use of this material in fabricating patient-specific flexible soft tissue implants with fine features, complex geometries, and relevant mechanical properties.

The SLA printer used on the production line was a Formlabs 3BL, a third-generation light-based printer designed for large-scale biomedical parts and medical devices. The printer features a 33.5 × 20 × 30 cm build volume for anatomically sized prints, a low force display unit for rapid printing, and automatic resin dispensing for efficient production. This efficient manufacturing process has enabled validation in multiple FDA-cleared workflows.

There have been few detailed reports on GMP-manufactured materials intended for implantation based on decellularized extracellular matrix [18, 19] or natural biomaterials [20]. However, none have classified the quality management process for the large-scale production of the implanted products for clinical approval. In line with our first objective, detailed documentation of the production process from build design, printing, inspection, release, and characterization was recorded for the prototyping of macroporous NAC implants (supplemental 1). Because the implants were acellular, shape and structure were key attributes to define acceptable process development criteria.

To validate our second objective, we classified process capabilities to identify minimum acceptable parameters based on physical, architectural, and visual inspection characteristics, thereby standardizing feature quality across future runs (table 1). Physical characteristics showed the least variability in the dimensional tolerance range, demonstrating the reproducibility of SLA technology. More variability was observed in architectural characteristics innate to NAC design and material characteristics. In piloting NAC production in a GMP facility, we identified preliminary inconsistencies and process limitations to guide future efforts to build risk management strategies around the technology.

Batch consistency was first established through consistent material mass among the samples (figure 3(A)). A key difference between F 80 A and E 50 A geometries was the variation in mechanical properties (figure 3(B)). Implants printed with E 50 A resin, which had a lower shore hardness, showed a closer resemblance to the mechanical properties of human tissue but more variation in the reproducibility of architectural features. In contrast, implants printed with F 80 A resin had a significantly higher modulus and higher consistency in the resolution of print features. However, the macroporous architecture in both designs significantly decreased the mechanical strength compared to the respective bulk material. Infill pattern, infill density, and strut diameter are useful parameters to modify for the prototyping of soft tissue implants that encourage tissue infiltration.

The Young’s Modulus of healthy soft tissues is in the range of 0.1 and 1000 kPa, depending on the tissue of origin [21]. The moduli of various components of human breast tissues have been quantified, including glandular tissue (11.28 ± 5.79 kPa), and fatty tissue (9.24 ± 4.48 kPa) [22]. Human nipple tissue has been reported to have a modulus of 257 ± 210 kPa (*n* = 3) [23]. The compressive modulus of porcine nipple teat, a similar structure to the human NAC, has previously been published at approximately 50 kPa [9]. Notably, the implants with macroporous structures in E 50 A geometries fell within the range of soft tissue, strengthening potential implications for the use of this material in soft tissue fabrication. E 0.5 NAC moduli values approached the upper end of published human NAC moduli values, measuring an average of 419.1 ± 90.2 kPa. Further iterations of design could lower the modulus while still maintaining structural integrity. Within macroporous structures, the compressive strength and modulus can be further fine-tuned by selecting an appropriate infill pattern [14, 24]. Thus, for soft tissue applications, it is desirable to minimize the scaffold volume by reducing material consumption during the printing process to mimic tissue mechanics. Macroporous geometries decreased the modulus significantly when compared to fully dense NACs. As the physiological environment is complex, future mechanical testing could include interface shear testing to account for twisting or sliding forces that the implant may encounter after implantation.

To complete our third objective, we assessed the consistency in patterned architecture, porosity, and filling efficiency of an acellular hydrogel. Additive manufacturing is a unique prototyping solution that allows for the simultaneous control of multiple design parameters, enabling an infinite number of design variants [25]. We employed brightfield and MicroCT imaging to validate the quality of the final product. The millimeter-scale size of the implant made it important to characterize architectural integrity to ensure consistency and reproducibility. We observed consistency in pore diameter within the base layer of the NAC implants, with more cosmetic artifacts in the E 50 A samples (figure 4(A)). Little variability was observed in all samples (figure 4(E)). Print integrity was also maintained throughout the height of the structure, with some defects observed in E 50 A geometries in higher layers. The apical layer was consistently thicker than the subsequent layers in E 50 A geometries (figure 5).

Because the E 50 A material is inherently softer than the F 80 A material, the thin features in E 50 A implants had less integrity than those in the F 80 A geometries. This resulted in increased porosity in the 0.4 mm geometry due to deformities originating in the center of the print, causing the warping of features that occupied less area in each layer (figure 6). To offset this, design adjustments such as increasing the strut diameter, changing the infill pattern, or increasing the infill density can reduce printing errors for this material. Precise control of pore size is critical for encouraging tissue ingrowth and neovascularization in implanted constructs. Larger pore sizes have proven to support soft tissue ingrowth and neovascularization [26, 27]. The balance between maintaining printability for successful prototyping and soft mechanical properties for physiological relevance must be optimized to produce a superior 3D printed NAC implant.

The non-degradable Formlabs elastomer is designed to provide stability and maintain non-planar geometrical architecture for long-term implantation. However, to encourage tissue integration and prevent the need for repeated surgical intervention, a biological material with extracellular matrix cues and cellular modularity is needed [28]. Here, we used the GelMA hydrogel due to its ease of tunability, cell binding motifs, and biodegradability [29]. Despite differences in pore area and porosity, all tested geometries supported hydrogel filling throughout the height of the print, with no leakage observed (figures 7(A) and (B)).

The regulatory pathway for the NAC remains under consideration. We anticipate that regulatory approval for the implants would follow either i) Class III medical device classification through pre-market approval assessment (PMA), or ii) Class II medical device classification through 510(k) pathway. If the NAC implant is deemed suitable for 510(k) pathway approval, substantial comparison to an existing device would be necessary for clearance. In this case, an approved device such as the Medpor implant for ear reconstruction can be used for comparison. If the PMA pathway is necessary, risk can be further mitigated through well-characterized manufacturing processes. The designated approval pathway will depend on the level of risk associated with factors such as the use of novel materials, the long-term implantation strategy, and the subcutaneous surgical technique.

Because GelMA and its photoinitiator have not been used in any FDA-approved products, regulatory approval would require additional testing to prove thorough biological evaluation, chemical characterization, degradation and sterilization, and long-term *in vivo* implantation studies following the appropriate ISO 10 993 family of standards. Alternatively, future production would consider replacing this material with an alternative soft material used in existing products, such as hyaluronic acid or collagen. These materials have been established in many FDA-approved products for dermal, orthopedic, and ophthalmic applications [30, 31].

Future production considerations should also include sterilization methods. Ethylene oxide or gamma/electron beam irradiation can be used to sterilize a printed NAC. Previous studies have verified that ethylene oxide sterilization does not affect the surface characteristics of E 50 A, while autoclaving alters the material surface from hydrophobic to hydrophilic [32]. If sensitive biological components (such as cells or growth factors) will be incorporated within the hydrogel component, each component should be sterilized separately and then combined under aseptic conditions to assemble a complete implant. Hydrogel components may also be sterilized via ethylene oxide or sterile filtered, depending on their sensitivity. Regulatory approval would require additional testing to prove thorough biological evaluation, chemical characterization, degradation and sterilization, and long-term *in vivo* implantation studies following the appropriate ISO 10 993 family of standards.

While macroporous architectures encourage tissue ingrowth and integration, they provide a challenging landscape while printing supported structures. The most significant limitation was identified in the printing of unbridged horizontal struts present in the grid infill design. As a result, several cosmetic defects were observed in the E 50 A geometries. This process informed measures to be taken in future runs to optimize geometric designs to support successful large-scale manufacturing. Other sources of failure were observed, including operator errors such as inadequate washing and inconsistencies in post-processing quality amongst technicians. In addition to grid, other infill geometries such as honeycomb, tetrahedral, and trabecular geometries were considered. However, the grid pattern was selected because of its superior post-processing performance, allowing for efficient removal of uncured resin. Additionally, its regular pore geometry enhanced manufacturing consistency and reproducibility. From this, key areas are identified to build process controls for regulated production. By piloting implant GMP production, we developed preliminary documentation and procedures to make SLA a viable option for reproducible NAC production.

## 5. Conclusion

Here, we established architectural and mechanical parameters for the large-scale manufacturing of SLA-printed NAC implants that resemble soft tissue. The macroporous architecture was designed to facilitate hydrogel infusion, delivering biological cues and nutrients to invading tissue. Mechanical properties showed resemblance to native tissue and were suturable for tissue implantation. Overall, SLA printing achieved consistency in patterned features throughout the height of the constructs. However, the prevalence of cosmetic defects was higher in E 50 A geometries. This work presents an overview of the manufacturing considerations for GMP production. Because the translation of 3D printed biological products has been limited, we sought to implement a standardized strategy in which the first step involves characterization of the implant’s physical properties. A rational next step toward translation would include a thorough evaluation of the biological response of the materials. Ongoing studies will investigate the *in vivo* response to the 3D printed NAC implants and quantify the extent of tissue ingrowth. While this process was carefully designed for the manufacturing of NACs for nipple reconstruction, it can be applied to various engineered soft tissue implants intended for long-term implantation to produce a reproducible and reliable product.

## Data Availability

All data that support the findings of this study are included within the article and any supplementary files. Supplementary documentation 1 available at https://doi.org/10.1088/1758-5090/ae7697/data1.

## References

[1] Mondschein R J, Kanitkar A, Williams C B, Verbridge S S, Long T E (2017). Polymer structure-property requirements for stereolithographic 3D printing of soft tissue engineering scaffolds. Biomaterials.

[2] Ligon S C, Liska R, Stampfl J, Gurr M, Mülhaupt R (2017). Polymers for 3D printing and customized additive manufacturing. Chem. Rev..

[3] Seet W T, Mat Afandi M A, Ishak M F, Hassan M N F, Ahmat N, Ng M H, Maarof M (2023). Quality management overview for the production of a tissue-engineered human skin substitute in Malaysia. Stem Cell Res. Ther..

[4] Aghayan H-R, Arjmand B, Norouzi-Javidan A, Saberi H, Soleimani M, Tavakoli S A-H, Khodadadi A, Tirgar N, Mohammadi-Jahani F (2012). Clinical grade cultivation of human Schwann cell, by the using of human autologous serum instead of fetal bovine serum and without growth factors. Cell Tissue Bank.

[5] Elliott M J (2017). Tracheal replacement therapy with a stem cell-seeded graft: lessons from compassionate use application of a GMP-compliant tissue-engineered medicine. Stem Cells Transl. Med..

[6] Stace E T, Dakin S G, Mouthuy P-A, Carr A J (2016). Translating regenerative biomaterials into clinical practice. J. Cell Physiol..

[7] Stratton S, Manoukian O S, Patel R, Wentworth A, Rudraiah S, Kumbar S G (2018). Polymeric 3D printed structures for soft-tissue engineering. J. Appl. Polym. Sci..

[8] Xia P, Luo Y (2022). Vascularization in tissue engineering: the architecture cues of pores in scaffolds. J. Biomed. Mater. Res. B.

[9] Van Belleghem S, Mahadik B, Snodderly K, Mote Z, Jiang B, Yu J R, McLoughlin S, He X, Nam A J, Fisher J P (2021). Dual extrusion patterning drives tissue development aesthetics and shape retention in 3D printed nipple-areola constructs. Adv. Healthcare Mater..

[10] Van Belleghem S, Torres L, Santoro M, Mahadik B, Wolfand A, Kofinas P, Fisher J P (2020). Hybrid 3D printing of synthetic and cell-laden bioinks for shape retaining soft tissue grafts. Adv. Funct. Mater..

[11] Zhang L, Fu L, Zhang X, Chen L, Cai Q, Yang X (2021). Hierarchical and heterogeneous hydrogel system as a promising strategy for diversified interfacial tissue regeneration. Biomater. Sci..

[12] Atala A, Kasper F K, Mikos A G (2012). Engineering complex tissues. Sci. Transl. Med..

[13] Xian G (2025). Gyroid-structured scaffolds guide uniform ossification and modulate vascular morphology during rat calvarial bone defect regeneration. J. Biomed. Mater. Res. A.

[14] Maevskaia E, Guerrero J, Ghayor C, Bhattacharya I, Weber F E (2023). Triply periodic minimal surface-based scaffolds for bone tissue engineering: a mechanical, *in vitro* and *in vivo* study. Tissue Eng. A.

[15] Shen M (2023). Bioceramic scaffolds with triply periodic minimal surface architectures guide early-stage bone regeneration. Bioact. Mater..

[16] U.S. Food and Drug Administration (2017). Technical considerations for additive manufactured medical devices—guidance for industry and food and drug administration staff. https://www.fda.gov/media/97633/download.

[17] Grab M, Jaud S, Thierfelder N, Hagl C, Wimmer B, Ahrens M, Stana J, Mela P, Grefen L (2025). Flexible 3D-printable materials for application in medical research: a comprehensive comparison of commercially available materials. 3D Print. Addit. Manuf..

[18] Prat-Vidal C (2020). First-in-human PeriCord cardiac bioimplant: scalability and GMP manufacturing of an allogeneic engineered tissue graft. eBioMedicine.

[19] Anderson A E (2022). An immunologically active, adipose-derived extracellular matrix biomaterial for soft tissue reconstruction: concept to clinical trial. npj Regen. Med..

[20] Sierra‐Sánchez Á (2020). Hyaluronic acid biomaterial for human tissue-engineered skin substitutes: preclinical comparative *in vivo* study of wound healing. J. Eur. Acad. Dermatol. Venereol..

[21] Arnold N, Scott J, Bush T R (2023). A review of the characterizations of soft tissues used in human body modeling: scope, limitations, and the path forward. J. Tissue Viab..

[22] Rzymski P, Skórzewska A, Skibińska-Zielińska M, Opala T (2011). Clinical research factors influencing breast elasticity measured by the ultrasound shear wave elastography—preliminary results. Arch. Med. Sci..

[23] Samadi A, Premaratne I D, Wright M A, Bernstein J L, Lara D O, Kim J, Zhao R, Bonassar L J, Spector J A (2021). Nipple engineering: maintaining nipple geometry with externally scaffolded processed autologous costal cartilage. J Plast. Reconstr. Aesthet. Surg..

[24] Saleh M, Anwar S, Al-Ahmari A M, Alfaify A (2022). Compression performance and failure analysis of 3D-printed carbon fiber/PLA composite TPMS lattice structures. Polymers.

[25] Morrison R J, Kashlan K N, Flanangan C L, Wright J K, Green G E, Hollister S J, Weatherwax K J (2015). Regulatory considerations in the design and manufacturing of implantable 3D-printed medical devices. Clin. Transl. Sci..

[26] Chimutengwende-Gordon M, Dowling R, Pendegrass C, Blunn G, Egles C (2018). Determining the porous structure for optimal soft-tissue ingrowth: an *in vivo* histological study. PLoS One.

[27] Mukasheva F, Adilova L, Dyussenbinov A, Yernaimanova B, Abilev M, Akilbekova D (2024). Optimizing scaffold pore size for tissue engineering: insights across various tissue types. Front. Bioeng. Biotechnol..

[28] Griveau L (2022). Design and characterization of an *in vivo* injectable hydrogel with effervescently generated porosity for regenerative medicine applications. Acta Biomater..

[29] Xiao S (2019). Gelatin methacrylate (GelMA)-based hydrogels for cell transplantation: an effective strategy for tissue engineering. Stem Cell Rev. Rep..

[30] Sen C K, Friday A, Khanna S, Roy S (2025). Collagen-based products in wound, skin, and health care. Adv. Wound Care.

[31] Granché R, Parekh K, Farjood E, Shriver S, McFarland R, Ying H, Kumar M, Addepalli P, Christ G J, Healy K E (2026). Interaction-driven classification of hyaluronic acid products. Nat. Rev. Bioeng..

[32] Dyner M (2025). Biocompatibility of materials dedicated to non-traumatic surgical instruments correlated to the effect of applied force of working part on the coronary vessel. Materials.

